# The Most Impactful Articles on Cauda Equina Syndrome

**DOI:** 10.7759/cureus.38069

**Published:** 2023-04-24

**Authors:** Aasim Hawa, Adwin Denasty, Karim Elmobdy, Addisu Mesfin

**Affiliations:** 1 Orthopedics, University of Rochester Medical Center, Rochester, USA

**Keywords:** bibliometrics analysis, top cited, impactful articles, spine conditions, cauda equina syndrome

## Abstract

Cauda equina syndrome (CES) is an uncommon condition that can lead to permanent neurological deficits if not diagnosed and addressed promptly. Varying prognoses, including retropulsed fracture fragments, disc herniations, and epidural abscesses, can result in CES. Our objective was to identify the top 50 most impactful articles on CES and analyze the characteristics of these publications. In August of 2021, we used the Web of Science Core Collection bibliographic database to query the phrase “cauda equina syndrome.” Articles between 1900 and 2021 were included in the search, and these articles were ranked based on the number of citations. The following variables were recorded: title, first author, journal, year of publication, number of citations, country of origin, the institution of publication, and topic of the paper. A total of 2096 articles matched the search criteria. The top 50 most impactful articles ranged from 43 to 439 in their number of citations. All articles on the list were published in English, with the year of publication ranging from 1938 to 2014. The United States accounted for the greatest number of articles published at 27. The medical journal Spine accounted for the greatest number of publications at nine. And the 2000s was the decade with the most cited articles. It is generally acknowledged that the clinical signals for CES are diverse with no predictive value on patient outcomes. Similar uncertainty exists in the etiology of the condition, though CES induced by spinal anesthesia is a factor of particular interest. Additionally, it is generally recognized that delayed diagnosis of the condition often results in permanent neurological deficits. Identification of the most impactful articles on CES is critical in drawing attention to this significant condition.

## Introduction and background

Cauda equina syndrome (CES) is a relatively rare condition affecting the nerve bundles towards the end of the spinal cord, and it requires urgent treatment to prevent rapid symptom progression [1]. Symptoms characteristic of this condition include acute or chronic lower back pain, numbness, weakness, sciatic nerve pain, loss of lower motor function and bladder, bowel, and sexual dysfunction, depending upon the rate of onset [2,3]. The term cauda equina anatomically refers to the spinal nerves L2-L5, S1-S5, as well as the coccygeal nerve [4]. Cauda equina syndrome is a result of the compression of some or all of these nerves [2]. The most common cause of CES is lumbar disc herniation, with 45% of all cases attributed to this condition [4]. Other causes include spinal stenosis, cysts, fractures, tumors, or any other lesions that can cause compression to the cauda equina [1]. Diagnosis of CES involves imaging via MRI or CT. Decompressive surgery, ideally within 48 hours of symptom onset, is the most common treatment option [4,5]. Treatment must begin urgently to prevent permanent neurological damage and to reverse any existing symptoms [1,3].

Identification articles discussing CES can be beneficial in enhancing the treatment of this surgical emergency, improving the care of patients with CES, and assessing the medico-legal implications of this condition. Our objective was to identify the most impactful articles on CES and to analyze the characteristics of these articles.

## Review

Methods

In August 2021, we used the Web of Science Core Collection bibliographic database to query the key phrase “cauda equina syndrome.” Articles between 1900 and 2021 were included in the search, and non-English literature was excluded. Search categories included clinical neurology, surgery, orthopedics, and more.

These articles were ranked based on the number of citations. We further sorted the results by excluding articles that did not solely focus on CES or were based on animal studies. Key information regarding each article was recorded, including title, first author, journal, year of publication, and the number of citations. Next, we listed the top 50 cited articles in rank order. The country of origin for each article was then recorded. In addition to these two lists, we sorted the articles by journal of publication, the decade of publication, the first author, the institution of origin, and the content of the article.

Results

Top 50 Cited Publications

A total of 2096 articles matched the search criteria. The top 50 most influential articles determined by the number of citations are listed in Table 1. These articles ranged from 43 to 439 in their number of citations, with a total number of 4406 citations among all papers. On average, each article had approximately 90 citations. Twelve publications had over 100 citations, with the most highly-cited article being *Cauda equina syndrome after continuous spinal anesthesia* by Rigler et al., published in 1991 [6]. The second-most cited paper was the work by Lambert et al. published in 1994, and the third in the number of citations was the article by Ahn et al. published in 2000 [7,8]. All articles on the list were published in English, with the year of publication ranging from 1938 to 2014.

**Table 1:** Top 50 cited publications listed as per rank order determined by the number of citations

Author	Year of Publication	Journal	Number of Citations	Title of Article
Rigler et al. [6]	1991	Anesthesia and Analgesia	439	Cauda equina syndrome after continuous spinal anesthesia
Lambert et al. [7]	1994	Anesthesiology	218	Irreversible conduction block in isolated nerve by high concentrations of local anesthetics
Ahn et al. [8]	2000	Spine	206	Cauda equina syndrome secondary to lumbar disc herniation: a meta-analysis of surgical outcomes
Assendelft et al. [9]	1996	Journal of Family Practice	174	Complications of spinal manipulation: a comprehensive review of the literature
Schildhauer et al. [10]	2006	Journal of Orthopaedic Trauma	168	Decompression and lumbopelvic fixation for sacral fracture-dislocations with spino-pelvic dissociation
Blau et al. [11]	1961	Lancet	155	Intermittent claudication of the cauda equina: an unusual syndrome resulting from central protrusion of a lumbar intervertebral disc
Kostuik et al. [12]	1986	The Journal of Bone and Joint Surgery	150	Cauda equina syndrome and lumbar disc herniation
Gleave et al. [13]	2002	British Journal of Neurosurgery	110	Cauda equina syndrome: what is the relationship between timing of surgery and outcome?
Gardner et al. [14]	2011	European Spine Journal	106	Cauda equina syndrome: a review of the current clinical and medico-legal position
Bellabarba et al. [15]	2006	Spine	105	Complications associated with surgical stabilization of high-grade sacral fracture dislocations with spino-pelvic instability
Fraser et al. [16]	2009	Archives of Physical Medicine and Rehabilitation	101	Cauda equina syndrome: a literature review of its definition and clinical presentation
Shapiro [17]	2000	Spine	101	Medical realities of cauda equina syndrome secondary to lumbar disc herniation
Drasner et al. [18]	1994	Anesthesiology	99	Persistent sacral sensory deficit induced by intrathecal local anesthetic infusion in the rat
Ehni [19]	1969	Journal of Neurosurgery	99	Significance of the small lumbar spinal canal: cauda equina compression syndromes due to spondylosis (Part 1: Introduction)
Shapiro [20]	1993	Neurosurgery	99	Cauda equina syndrome secondary to lumbar disc herniation
Ross et al. [21]	1992	Regional Anesthesia	96	Local anesthetic distribution in a spinal model: a possible mechanism of neurologic injury after continuous spinal anesthesia
Lambert et al. [22]	1991	Anesthesia and Analgesia	93	Cauda equina syndrome and continuous spinal anesthesia
Wilson [23]	1969	Journal of Neurosurgery	86	Significance of the small lumbar spinal canal: cauda equina compression syndromes due to spondylosis (Part 3: Intermittent Claudication)
Drasner et al. [24]	1992	Anesthesiology	79	Cauda equina syndrome following intended epidural anesthesia
McCarthy et al. [25]	2007	Spine	79	Cauda equina syndrome: factors affecting long-term functional and sphincteric outcome
DeLong et al. [26]	2008	Journal of Neurosurgery: Spine	77	Timing of surgery in cauda equina syndrome with urinary retention: meta-analysis of observational studies
Orendacova et al. [27]	2001	Progress in Neurobiology	76	Cauda equina syndrome
Tatter et al. [28]	1994	Journal of Neurosurgery	75	Hemorrhage into a lumbar synovial cyst causing an acute cauda equina syndrome: case report
Delamarter et al. [29]	1991	Spine	72	1991 Volvo Award in Experimental Studies – Cauda equina syndrome: neurologic recovery following immediate, early or late decompression
Qureshi et al. [30]	2007	European Spine Journal	70	Cauda equina syndrome treated by surgical decompression: the influence of timing on surgical outcome
Epstein et al. [31]	1964	American Journal of Roentgenology Radium Therapy and Nuclear Medicine	67	The effect of anatomic variations in lumbar vertebrae and spinal canal on cauda equina and nerve root syndromes
Todd [32]	2005	British Journal of Neurosurgery	66	Cauda equina syndrome: the timing of surgery probably does influence outcome
Schoenecker et al. [33]	1990	The Journal of Bone and Joint Surgery	64	Cauda equina syndrome after in situ arthrodesis for severe spondylolisthesis at the lumbosacral junction
Bell et al. [34]	2007	British Journal of Neurosurgery	63	Cauda equina syndrome - What is the correlation between clinical assessment and MRI scanning?
Gerancher [35]	1997	Anesthesiology	61	Cauda equina syndrome following a single spinal administration of 5% hyperbaric lidocaine through a 25-gauge Whitacre needle
Balasubramanian et al. [36]	2010	British Journal of Neurosurgery	61	Reliability of clinical assessment in diagnosing cauda equina syndrome
Kennedy et al. [37]	1999	European Spine Journal	61	Predictors of outcome in cauda equina syndrome
Shephard [38]	1959	British Medical Journal	59	Diagnosis and prognosis of cauda equina syndrome produced by protrusion of lumbar disk
Lavy et al. [39]	2009	British Medical Journal	59	Cauda equina syndrome
Kohles et al. [40]	2004	Spine	56	Time-dependent surgical outcomes following cauda equina syndrome diagnosis: comments on a meta-analysis
Prusick et al. [41]	1988	The Journal of Bone and Joint Surgery	53	Cauda equina syndrome as a complication of free epidural fat-grafting: a report of two cases and a review of the literature
Bartleson et al. [42]	1983	Annals of Neurosurgery	53	Cauda equina syndrome secondary to long-standing ankylosing spondylitis
Russell et al. [43]	1973	Annals of Internal Medicine	52	The cauda equina syndrome of ankylosing spondylitis
Haldeman et al. [44]	1992	Spine	51	Cauda equina syndrome in patients undergoing manipulation of the lumbar spine
Nielsen et al. [45]	1980	Urologia Internationalis	51	A urodynamic study of cauda equina syndrome due to lumbar disc herniation
Ferguson et al. [46]	1938	British Journal of Surgery	49	Paralysis of the bladder and associated neurological sequelae of spinal anaesthesia (cauda equina syndrome)
Diaz [47]	2002	Anesthesiology	48	Permanent paraparesis and cauda equina syndrome after epidural blood patch for postdural puncture headache
Hellstrom et al. [48]	1986	Journal of Urology	48	Late urodynamic findings after surgery for cauda equina syndrome caused by a prolapsed lumbar intervertebral disk
Lisai et al. [49]	2001	Spine	46	Cauda equina syndrome secondary to idiopathic spinal epidural lipomatosis
Loo et al. [50]	1999	Acta Anaesthesiologica Scandinavica	45	Cauda equina syndrome after spinal anaesthesia with hyperbaric 5% lignocaine: a review of six cases of cauda equina syndrome reported to the Swedish Pharmaceutical Insurance 1993-1997
Chau et al. [51]	2014	World Neurosurgery	45	Timing of surgical intervention in cauda equina syndrome: a systematic critical review
Olivero et al. [52]	2009	Journal of Spinal Disorders & Techniques	45	Cauda equina syndrome (CES) from lumbar disc herniations
Spector et al. [2]	2008	Journal of the American Academy of Orthopaedic Surgeons	44	Cauda equina syndrome
Brown et al. [53]	2001	Spine	44	Surgery for lumbar disc herniation during pregnancy
Dosoglu et al. [54]	2001	European Spine Journal	43	Posterior epidural migration of a lumbar disc fragment causing cauda equina syndrome: case report and review of the relevant literature

Countries of Origin

Twelve countries were represented as countries of origin for these 50 articles (Table 2). The United States accounted for the greatest number of articles at 27. The United Kingdom produced 12, followed by Canada at two. The remaining represented countries produced one article each.

**Table 2:** Countries of origin of the top 50 cited publications

Country	Number of Articles
United States of America	27
United Kingdom	12
Canada	2
Australia	1
Denmark	1
Finland	1
Ireland	1
Italy	1
Netherlands	1
Slovakia	1
Sweden	1
Turkey	1

Top Journals of Publication

Of all the journals represented in the top 50 list, Spine published the greatest number of articles with nine (Table 3). *Anesthesiology* accounted for the second-greatest number of publications at five. British Journal of Neurosurgery, European Spine Journal, and Journal of Neurosurgerypublished four articles each. Three more destination journals produced multiple publications.

**Table 3:** Most common journals that feature the top 50 cited articles

Journal	Number of Articles
Spine	9
Anesthesiology	5
British Journal of Neurosurgery	4
European Spine Journal	4
Journal of Neurosurgery	4
The Journal of Bone and Joint Surgery	3
Anesthesia and Analgesia	2
British Medical Journal	2

Decades of Publication

The 2000s was the most active decade in publishing articles on CES (20 articles), followed closely by the 1990s (15 papers published) as shown in Table 4. The oldest of the articles,* Paralysis of the bladder and associated neurological sequelae of spinal anaesthesia (cauda equina syndrome)* by Ferguson et al., was published in 1938 [46]. The most recent publication is the 2014 article by Chau et al. titled T*iming of surgical intervention in cauda equina syndrome: a systematic critical review* [51].

**Table 4:** Most common decades of publication for the top 50 cited articles

Decade	Number of Articles
2000s	20
1990s	15
1980s	5
1960s	4
2010s	3

Top Authors

Only two first authors produced multiple publications: Drasner [18,24], and Shapiro [17,20] (Table 5). Both of these authors published two articles each. The remaining 46 articles were published by unique first authors.

**Table 5:** Authors with multiple first authorships in the top 50 cited publications

First Author	Number of Articles
Drasner K	2
Shapiro S	2

Top Institutions of Publication

A total of 42 institutions were affiliated with the top 50 articles (Table 6). Five institutions, four of which are located in the United States while one is located in the United Kingdom, accounted for multiple articles. The University of California San Francisco contributed the greatest number of publications at four. The University of Washington followed with three publications. Each of the following institutions was credited with two publications: Brigham and Women’s Hospital, University Hospitals of Leicester, and Wishard Memorial Hospital.

**Table 6:** Most common institutions of publication for the top 50 cited articles

Institution	Location	Number of Articles
University of California San Francisco	San Francisco, California, USA	4
University of Washington	Seattle, Washington, USA	3
Brigham and Women’s Hospital	Boston, Massachusetts, USA	2
University Hospitals of Leicester	Leicester, England, UK	2
Wishard Memorial Hospital	Indianapolis, Indiana, USA	2

Paper Topics

The top 50 cited articles were characterized into the following topics: disc herniations, anesthetic complications, the timing of surgical intervention, fractures/dislocations, and ankylosing spondylosis (Table 7). The most common topics were disc herniations and anesthetic complications, with 11 articles falling into each category. The next most-frequent topic was the timing of surgical interventions with six publications, followed by two each on fractures/dislocations and ankylosing spondylosis.

**Table 7:** Most common paper topics for the top 50 cited articles

Article Type	Number of Articles
Disc Herniations	11
Anesthetic Complications	11
Timing of Surgical Intervention	6
Fractures/Dislocations	2
Ankylosing Spondylosis	2

Discussion

Cauda equina syndrome is a rare but serious condition resulting from the compression of specific sacral and lumbar nerve roots [39]. Due to the relatively low incidence of this condition, the pathophysiology of CES remains unclear, and diagnosis remains challenging due to a lack of clarity in initial symptoms and their progression [2]. It is estimated that clinical diagnosis of CES is associated with a false positive rate of 43% [1]. No consensus for the exact symptoms of this condition has been reached, though CES commonly presents with sensory loss and bladder dysfunction, with the latter widely regarded as the most important factor to consider for diagnosis [55,56]. Additionally, symptoms such as back pain, bladder dysfunction, and weakness can often present under differing timelines, ranging from progressing rapidly over days to prolonged development over months [55]. At its 2012 annual general meeting, the British Association of Spine Surgeons discussed the diagnosis and treatment of CES. The members agreed that clinical signals for CES are diverse with no predictive value of these signs on patient outcomes [57].

The etiology of CES is also quite diverse, with causes ranging from disc degeneration to infection. The most common cause of CES is lumbar disc herniation [4]. Additionally, CES can be induced operatively, through injury of the nerve roots during surgery [55]. Complications associated with spinal manipulation therapies can also lead to CES, as discussed in the article by Assendelft et al. identified in this bibliometrics analysis [9]. Further, it appears that CES following spinal anesthesia is an etiological factor of special consideration, as 11 of the 50 articles listed in this study discuss anesthetic-induced CES (Table 7).

Treatment of CES most commonly involves surgical decompression, though some concerns about the efficacy of surgical treatment have been raised [13]. Additionally, the urgency of surgical decompression is controversial. It has generally been recommended that CES is treated with urgent surgery; though, some have argued that delays in surgery may be beneficial [57,58]. 

Due to the diversity in the development, presentation, and treatment of CES, an understanding of the top 50 cited articles discussing this condition can provide insights. The most highly cited article addressing CES is by Rigler et al. from 1991 [6]. This publication discusses four cases of CES in patients following continuous spinal anesthesia. Each patient was scheduled for a different operation, and various types and gauges of needles and catheters were utilized. Though there was diversity in the exact circumstances of each case, all involved the development of CES following spinal anesthetics. In all cases, a focal sensory block was indicated, suggesting maldistribution of the anesthetic. Additionally, a relatively high dose of the anesthetic was administered in all four patients. The authors propose a ceiling to local anesthetic dose as a potential solution for this complication [6].

The publication with the second most citations is the 1994 article titled I*rreversible conduction block in isolated nerve by high concentrations of local anesthetics *[7], an immediate extension of the aforementioned most highly cited article. This publication aims to explain the development of CES in the four patients discussed previously [6]. Specifically, this study explores the observed conduction blocks present following anesthesia. Desheathed frog sciatic nerves were isolated and exposed to varying concentrations and types of local anesthetics. It was observed that irreversible total conduction blockade was achieved in nerves exposed to 5% lidocaine or 0.5% tetracaine, whereas lower concentrations of these drugs only resulted in a partial block. The authors concluded that these local anesthetics were likely responsible for causing irreversible conduction block in the four cases of CES [7].

The 2000 article by Ahn et al. has the third most citations [8]. This study analyzes surgical outcomes of CES secondary to lumbar disc herniation, with a particular focus on clinical outcomes of patients with surgical decompression at varying lengths following the onset of symptoms. Forty-two citations met the inclusion criteria for this meta-analysis, translating to a total of 322 patient outcomes evaluated. There was no significant difference indicated in clinical outcome between patients treated with surgical intervention less than 24 hours post-CES onset, and those undergoing decompressive surgery between 24 and 48 hours following symptom onset. However, the study did find a significant difference in sensory, urinary, and rectal function in patients treated within 48 hours compared with those who were operated on following this 48-hour cutoff [8].

The oldest of the top 50 articles titled *Paralysis of the bladder and associated neurological sequelae of spinal anaesthesia (cauda equina syndrome)*, was published in 1938 [46]. This paper reports multiple cases in which patients developed symptoms consistent with CES following spinal anesthesia. Duracaine was specifically utilized as the anesthetic in all 14 of the cases, leading the authors to recommend the discontinuation of heavy use of this drug to achieve analgesia in patients [46].

The most recently published article is the work by Chau et al. [51]. This article, published in 2014, discusses the timing of surgical decompression in patients presenting with CES [51]. Specifically, the relevance of the established time cutoff of 48 hours was assessed, and a temporal ceiling was also analyzed in the work by Ahn et al. [8]. A literature review was conducted with five articles indicating analytical data relevant to this investigation. The authors found no evidence to support the merit of this highly-cited 48-hour cutoff; instead, they recommend surgical intervention “at the earliest practical opportunity.” The authors also conclude that many of the articles on this subject provide limited data and lack statistical significance to govern the treatment ideals of CES [51].

Another article in the top 50 bearing particular magnitude is the work by Gleave et al. [13]. This publication was one of the first to provide guidelines on categories of CES. The authors defined two subclasses of CES: incomplete cauda equina syndrome (CESI) and cauda equina syndrome with retention (CESR). Incomplete cauda equina syndrome is designated as CES with urinary complications, but in the absence of established urinary retention. On the other hand, CESR is defined as CES where the bladder is not under executive control, resulting in urinary retention and overflow incontinence. This highly-cited article presents definitions of the subcategories of CES [13]. 

Lastly, some of the recurring topics in these articles need to be mentioned. One of the most commonly explored subjects within clinical studies, prompting the creation of a separate category in Table 7, is CES induced by anesthetic complications. Cauda equina syndrome secondary to ankylosing spondylitis is also a subject discussed in multiple articles (Table 7). The importance of the timing of surgical intervention is another topic of interest, likely due to its controversy. Within the top 50 cited articles, some conclude that patient outcome is time-dependent [8,26,32], while others establish no significant correlation between clinical success and the timing of the surgery [30,51]. It is evident that controversy regarding this issue continues to exist. Another significant topic within the top 50 cited articles is the potential for predictors of clinical outcomes, especially given the incertitude of the relevance of symptomatic signals discussed earlier [57]. The article by McCarthy et al. was unable to find a correlation between various clinical factors and patient outcomes [25]. Kennedy et al. similarly could not obtain a significant enough correlation to establish definitive predictors of clinical success; though the authors did note that early diagnosis increased the probability of a positive outcome [37].

The primary limitation of this study is the fluid nature of citations that could impact the order of the top 50 cited articles. The database was searched in August 2021, and it is likely that the citations may change at a future date. However, dramatic trend changes in the citations are unlikely. Another limitation is that only the English language was included in the search criteria, so highly-cited articles in other languages may have been excluded.

## Conclusions

In conclusion, this study evaluated the most highly cited articles discussing CES. The United States produced the majority of these top 50 cited articles, with *Spine *publishing the greatest number of articles. Most of the articles were published in the 2000s, and a majority of the articles discussed CES in relation to disc herniations or anesthetic complications. The lack of clarity in pathophysiology, diagnosis, and treatment of this condition is evident upon analysis of the top cited articles on the syndrome. Controversy continues to exist regarding symptom presentation, clinical outcome predictors, and the efficacy of surgical intervention even among the most impactful articles on CES. 
